# Efficacy of artemether-lumefantrine, the nationally-recommended artemisinin combination for the treatment of uncomplicated falciparum malaria, in southern Laos

**DOI:** 10.1186/1475-2875-11-184

**Published:** 2012-06-08

**Authors:** Mayfong Mayxay, Maniphone Khanthavong, Odai Chanthongthip, Mallika Imwong, Tiengkham Pongvongsa, Bouasy Hongvanthong, Samalane Phompida, Viengxay Vanisaveth, Nicholas J White, Paul N Newton

**Affiliations:** 1 Wellcome Trust-Mahosot Hospital-Oxford University Tropical Medicine Research Collaboration, Mahosot Hospital, Vientiane, Lao PDR; 2 Faculty of Postgraduate Studies, University of Health Sciences, Vientiane, Lao PDR; 3 Centre for Clinical Vaccinology and Tropical Medicine, Churchill Hospital, University of Oxford, Oxford, UK; 4 Centre of Malariology, Parasitology and Entomology, Vientiane, Lao PDR; 5 Department of Molecular Tropical Medicine and Genetics, Faculty of Tropical Medicine, Mahidol University, Bangkok, Thailand; 6 Savannakhet Provincial Malaria Station, Savannakhet Province, Lao PDR; 7 Faculty of Tropical Medicine, Mahidol University, Bangkok, Thailand

**Keywords:** Clinical trial, *Plasmodium falciparum*, Malaria, Artemisinin-based combination therapy (ACT), Artemether-lumefantrine, Coartem, Laos

## Abstract

**Background:**

The Lao Government changed the national policy for uncomplicated *Plasmodium falciparum* malaria from chloroquine to artemether-lumefantrine (AL) in 2005. Since then, no information on AL efficacy has been reported. With evidence of resistance to artemisinin derivatives in adjacent Cambodia, there has been a concern as to AL efficacy. Monitoring of AL efficacy would help the Lao Government to make decisions on appropriate malaria treatment.

**Methods:**

The efficacy of a three-day, twice daily oral artemether-lumefantrine for the treatment of uncomplicated falciparum malaria in Xepon District, Savannakhet Province, southern Laos was studied over 42 days follow-up. This was part of a trial of thiamin supplementation in falciparum malaria.

**Results:**

Of 630 patients with *P. falciparum* enrolled in the trial of thiamin treatment, 549 (87%, 357 children ≤15 years and 192 adults) were included in this study. The per protocol 42-day cure rates were 97% (524/541) [96% (337/352) for children and 99% (187/189) for adults, p = 0.042]. By conventional intention-to-treat analysis, the 42-day cure rates adjusted for re-infection, were 97% (532/549) [96% (342/357) in children and 99% (190/192) in adults, p = 0.042]. The proportion of patients who remained parasitaemic at day 1 after treatment was significantly higher in children [33% (116/356)] compared to adults [15% (28/192)] (p < 0.001) and only one adult patient had detectable parasitaemia on day 2. There were no serious adverse events. Potential side effects after treatment were reported more commonly in adults (32%) compared to children (15%) (p < 0.001). Patients with recrudescent infections were significantly younger, had longer mean time to fever clearance, and had longer median time to parasite clearance compared to those who were cured.

**Conclusions:**

The current nationally-recommended anti-malarial treatment (artemether-lumefantrine) remains highly efficacious for the treatment of uncomplicated falciparum malaria five years after introduction in Laos. Regular monitoring is required in case artemisinin-resistant *P. falciparum* parasites should appear.

**Trial registration:**

ISRCTN85411059.

## Background

Although the incidence of *Plasmodium falciparum* malaria has declined recently in northern and central Lao PDR (Laos), malaria remains an important cause of morbidity and mortality in southern Laos, with an estimated incidence of 4.7 to 23.5/1,000 population between 2006-2008 [1-3]. The change of the Lao Government national treatment policy for uncomplicated falciparum malaria from chloroquine to artemether-lumefantrine in 2005 is likely to have contributed substantially to the reduction in the incidence of malaria in northern and central Laos. This change was prompted by high levels of *P. falciparum* resistance to chloroquine (CQ) and sulphadoxine-pyrimethamine (SP) [4-8]. The use of artemisinin combination therapy (ACT), artesunate + mefloquine, artemether-lumefantrine (AL) and dihydroartemisinin-piperaquine, has been shown to provide high cure rates (42-day failure rate of ≤ 6%) and good tolerability in Laos [1,9-12].

However, artemisinin resistance has emerged in western Cambodia [13-15], raising serious concerns that resistance could spread the ~ 400 km to southern Laos. In 2002-3, the 42-day cure rate following full treatment with AL was 97% in southern Laos despite a low-fat diet (which reduces lumefantrine absorption) and consequent low day 7 plasma lumefantrine concentrations [9]. AL has to be taken twice a day, and should be taken with fats [16-20] which may compromise adherence and efficacy respectively, so there is concern that AL efficacy could decline, particularly if protection from the co-administered artemether is compromised by emerging resistance.

Artemisinin resistance is manifest by a slowing in parasite clearance rates [15,21]. Reassuringly, of 861 patients with falciparum malaria recruited to three trials in southern Laos treated with ACT between 2002 and 2008, only 2 (0.23%) patients had parasite clearance times (PCT) longer than three days and 30 (3.48%) had PCT longer than 2 days [22]. To determine whether there has been any recent change, the efficacy of the nationally recommended ACT (3-day oral artemether-lumefantrine) for the treatment of uncomplicated falciparum malaria was reported. This was part of a large trial of thiamin treatment in *P. falciparum* malaria in southern Laos, which will be reported separately.

## Methods

### Study site, patients, clinical and laboratory procedures

The study was conducted between June and November from 2008-2010 at Xepon (30 beds) Inter-District Hospital (16.69^0^ N, 106.20^0^ E, 208 metres above sea level), Savannakhet Province, ~665 km southeast of Vientiane, the capital of Laos. Xepon (88 villages, population 48,000) is inhabited predominantly by rice farmers of the Lao Theung ethnic groups. Malaria transmission is seasonal with a peak during the rainy months of July and August [22].

The clinical trial of thiamin supplementation in falciparum malaria conducted among patients with *P. falciparum* malaria receiving anti-malarial therapy according to national guidelines, was an exploratory, double-blind, parallel group, placebo-controlled, randomized (variable blocks), superiority trial to compare the frequency of clinical adverse effects and thiamin status, during 42 days follow up, of either daily oral thiamin supplementation ([oral thiamin (5 mg tablet) (Olan-Kemed Co.Ltd, Bangkok, Thailand)] 10 mg immediately after anti-malarial drugs, followed by 10 mg daily for 7 days followed by 5 mg daily until day 42), or an identical placebo.

Patients were included in the trial of thiamin treatment provided that they or their guardians (in the case of children) gave fully informed written consent, were of any age, had microscopically confirmed *P. falciparum* infection or mixed *Plasmodium* species infections, with a history of fever, were willing and able to comply with the study protocol for the duration of the 42 days follow up, and had not taken a full course of any anti-malarial drugs in the previous three days. Patients were excluded if they had a history of hypersensitivity to thiamin or artemether-lumefantrine, presented with intercurrent non-malarial illness or any condition, which in the judgement of the investigator would place the subject at undue risk or interfere with the results of the study, and if they had clinically apparent suspected thiamin deficiency (beriberi) [23]. Patients included in the analysis for this paper were only those without danger signs or severe falciparum malaria [24], with *P. falciparum* mono- or mixed infections with other species, but an asexual *P. falciparum* parasite density of <250,000/μL, and were able to swallow artemether-lumefantrine at presentation.

Patients’ admission clinical details including current and past medical history, pre-treatment, and findings from physical examination were recorded on the case record form. Venous blood samples were taken for parasite counts, haematocrit, blood glucose, and lactate, and three blood spots were collected on 3MM filter paper (Whatman, Maidstone, UK) and stored in a plastic bag with silica gel for later malaria genotype analysis [22,25]. For women of child bearing age a urinary pregnancy test was performed before anti-malarial treatment was prescribed.

If the study criteria were met, patients were admitted to the hospital and treated with the nationally recommended anti-malarial - artemether (20 mg)-lumefantrine (120 mg) (Co-artem®, Novartis) provided by the Global Fund to fight AIDS, Tuberculosis and Malaria: one dose twice daily for three days. Dosing by body weight were one tablet if <15 kg, two tablets if 15–24 kg, three tablets if 25–34 kg, and four tablets if ≥35 kg. All patients were asked to take fatty food with the anti-malarial drugs and drug administration was directly observed by a study nurse. For children who could not swallow tablets, the appropriate drug dose was crushed and mixed with water and given in a syringe. If vomiting occurred within 30 minutes of anti-malarial administration, the full dose was repeated. Patient who vomited within 30 minutes to one hour, a half dose was re-administered. Patients who vomited >2 times within one hour were given the rescue treatment (artesunate 2.4 mg/kg IV stat, followed by 2.4 mg/kg IV at 12 hours and 24 hours and then daily until able to take oral medication).

Vital signs (axillary temperature, pulse, respiratory rate, and blood pressure) were measured every six hours. Patients and parasite counts were reviewed daily until two consecutive negative blood smears, then weekly for 42 days from the start of treatment or at other times if they felt unwell. Patients were discharged only when their fever and parasitaemia had cleared (defined as axillary temperature <37.5^0^ C and <1 parasite per 500 white cells on thick film after 2 negative slides, respectively) and were asked to return to the hospital at days 7, 14, 21, 28, 35 and 42 (or at any time the patients feel unwell) or were visited at home in case the patients did not return as planned.

At each weekly visit, the symptoms of the patients during the previous week were recorded. Finger-prick blood samples were taken for malaria blood smears and haematocrit and blood spots were collected onto filter paper strips from all patients with recurrent fever or malaria symptoms. PCR amplification was performed on paired samples for parasite genotyping to distinguish between reinfection and recrudescence using three parasite loci (MSP-1, MSP-2, and GLURP) [25]. The PCR tests were performed at the MORU malaria molecular laboratory, Bangkok, Thailand.

Patients with *P. falciparum* recurrence were retreated with artesunate 4 mg/kg/day in a single daily dose for 3 days (Day 0-2) (Guilin Pharmaceutical Co., Guilin, PRC) plus mefloquine 15 mg base/kg on day 1 and 10 mg base/kg on day 2 in single daily dose (Lariam^TM^, Roche Co, Switzerland). Those who had *P. vivax* appearance during follow up were treated with chloroquine (25 mg base/kg) for three days and further followed up. The incidence of any adverse event was recorded. Written informed consent was obtained from all participants.

Ethical clearance for the study was granted by the Lao National Ethics Committee for Health Research and the Oxford University Tropical Medicine Research Ethics Committee (OXTREC). The trial is registered with the ISRCTN via the University of Oxford as ISRCTN: 85411059. The trial was monitored by the Clinical Trials Support Group of Mahidol-Oxford Tropical Medicine Research Unit, Bangkok, Thailand.

### Outcome measures

The primary objective of the thiamin treatment trial was to determine whether the frequency of adverse events, after anti-malarial therapy, were significantly lower in those who received thiamin supplementation in comparison to those who did not. Here the PCR-corrected adequate clinical and parasitological responses at day 42 [26], the PCT (the time in hours from the first treatment dose to the first of two consecutive thick films that were negative for asexual falciparum parasites after checking ≥500 oil fields) and fever clearance times (FCT, time in hours from the start of treatment at which the tympanic temperature first dropped below 37.5^0^ C and remained below 37.5^0^ C for 48 hours), gametocytaemia, the frequency of adverse events, and changes in haematocrit following anti-malarial treatment are described.

### Statistical analysis

Data were analysed using Stata v9 (StataCorp, College Station, TX, USA). Comparisons between two groups were made by the Mann-Whitney *U*, Student’s *t*, chi-square, and Fisher’s exact tests, as appropriate. Cure rates were calculated as the proportion of patients with PCR-confirmed recrudescence using intention-to-treat (ITT) and per-protocol (PP) populations and by Kaplan-Meier survival analysis. In the ITT population all losses to follow-up were treated as failures and in the PP population losses to follow-up were excluded from the analysis. Patients with new infections were regarded as cures in both analyses. Gametocyte carriage was summarized as person-gametocytes-week rates calculated as the total number of weeks with gametocytes divided by the total number of weeks of follow-up. To identify independent predictors of treatment response, a stepwise logistic regression was constructed with recrudescence as the dependent variable and sex, age, admission symptoms (headache, chill, myalgia, dizziness, vertigo, nausea, vomiting, abdominal pain, diarrhea, anorexia, cough, dyspnea, and weakness) and signs (drowsiness, splenomegaly, and hepatomegaly), gametocyte carriage at admission, admission temperature, baseline haematocrit, fever and parasite clearance times, past history of malaria, and *P. vivax* appearance during follow up included as covariates. Only factors that were significant at p < 0.05 were retained in the final model.

## Results

**Figure 1:**
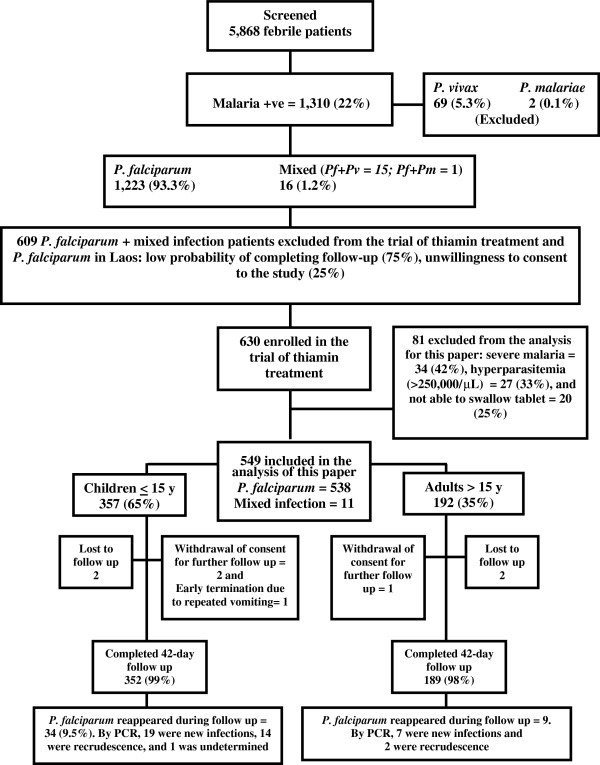
Patient flow diagram.

Of the 630 patients enrolled, 549 (87%; 276 randomized to thiamin and 273 to identical placebo) were included in the analysis for this paper. Patients were excluded (N = 81) if they had either severe disease [24] (n = 34) or parasitaemia >250,000/μL (n = 27) or were not able to swallow tablets on admission (n = 20) (see above, Figure 1). As the median (range) PCT (days) and mean (95%CI) FCT (hours) did not differ significantly between patients with and without thiamin supplementation [1 (1-3) vs 1 (1-3), p = 0.87; and 26.6 (24.7–28.6) vs 26.4 (24.4-28.4), p = 0.85, respectively] all 549 patients were included in a single group. Admission and follow-up haematocrit and gametocytaemia also did not significantly differ between the two trial groups (p > 0.05).

Of the 549 patients (Figure 1), 538 (98%) had *P. falciparum* mono-infection and 11 (2%) had mixed infections (10 *P. falciparum* + *P. vivax* and 1 *P. falciparum* + *P. malariae*), and 357 (65%) were children aged ≤15 years (Table 1). One child with persistent vomiting after taking AL was given parenteral artesunate and excluded from the study. Four patients (two children and two adults) were lost to follow up and three (two children and one adult) withdrew their consent for further follow up. Therefore, 352 (99%) and 189 (98%) children and adults, respectively, completed 42 days follow up (Figure 1).

**Table 1:** Admission demographic, clinical and laboratory details for patients included in the analysis of the efficacy of artemether-lumefantrine (Coartem™) for the treatment of uncomplicated falciparum malaria in Laos

**Variables**	**Age groups**		
	**All (n = 549)**	**Children (≤ 15 yrs) (n = 357)**	**Adults (> 15 yrs) (n = 192)**
Sex, M, no (%)	276 (50)	174 (49)	102 (53)
Age, years, median (range)	10 (0.5 - 73)	6 (0.5 - 15)	25 (16 – 73)
Body weight, kg	29.5 (28.0 – 30.9)	18.8 (17.8 – 19.8)	49.3 (48.2 – 50.4)
Height, cm	124.5 (122.0 –126.9)	108.4 (106.0 – 110.7)	154.4 (153.3 – 155.5)
Previous malaria attack, no. (%) of patients^a^	151 (27.5)	89 (25)	62 (32)
Axillary temperature, °C	38.4 (38.3 - 38.5)	38.5 (38.4 - 38.7)*	38.2 (38.0 - 38.3)
Patients without fever on admission, no. (%)	147 (27)	87 (24)	60 (31)
Systolic blood pressure, mm Hg	101.9 (100.8 -103.1)	95.1 (94.2 - 96.1)*	114.6 (113.0 - 116.1)
Diastolic blood pressure, mm Hg	69.7 (68.8 – 70.6)	64.6 (63.7 - 65.5)*	79.3 (78.2 - 80.4)
Pulse, beats/min	95.1 (94.0 - 96.2)	100.4 (99.0 -101.8)*	85.3 (84.4 – 86.3)
Respiratory rate/min	27.5 (27.0 – 28.1)	29.7 (29.0 – 30.3)*	23.5 (22.9 – 24.0)
Splenomegaly, no. (%) of patients	140/547 (26)	123/355 (35)*	17/192 (9)
Hepatomegaly, no (%) of patients	116/547 (21)	112/355 (31.5)*	4/192 (2)
Lymphadenopathy, no (%) of patients	2/545 (0.4%)	2/356 (0.6%)	0
Parasitaemia, geometric mean parasites/μL (95%CI)	19,775 (17,244 – 22,678)	27,241* (23,281 – 31,875)	10,902 (8,567 – 13,872)
Mixed malaria species infection, no.(%)	11 (2)	8 (2)	3 (1.5)
Gametocytaemia, no. (%) of patients	23 (4)	19 (5)	4 (2)
Gametocytaemia, geometric mean parasite/μL (95%CI)	216 (110 – 423)	201 (97 – 415)	328 (13 – 8,195)
Haematocrit, %	35.8 (35.2 – 36.3)	33.7 (33.1 – 34.4)*	39.5 (38.7 – 40.4)
Glucose, mmol/L	6.09 (5.97 – 6.20)	6.09 (5.93 – 6.24)	6.08 (5.90 – 6.27)
Lactate, mmol/L	2.9 (2.8 – 3.0)	3.1 (2.9 – 3.2)*	2.8 (2.6 – 2.9)

**NOTE.** Data are presented as mean values (95% CI), unless otherwise indicated.

* Significantly different from the other group (p < 0.001).

^a^ Defined as patient or patient’s guardian reporting that the patient had had a febrile illness with a positive malaria slide.

### Demographic and clinical details

All patients had a history of fever before admission and 402 (73%) were febrile at the time of enrollment. The proportion of patients without documented fever on admission was similar between children and adults (Tables 1 and 2). The admission mean temperature and geometric mean parasitaemia were significantly higher in children compared to adults, as were the proportions of patients with nausea, vomiting, irritability, or a palpable spleen or liver at presentation. The frequencies of headache, chill, myalgia, tinnitus, palpitations, and dyspnoea were significantly lower in children compared with adults (Table 2).

**Table 2:** Admission symptoms and signs for patients included in the analysis of the efficacy of artemether-lumefantrine (Coartem™) for the treatment of uncomplicated falciparum malaria in Laos

**Symptoms and signs**	**Total (n = 549)**	**Age groups**		
		**Children (≤ 15 years) (n = 357)**	**Adults (> 15 years)(n = 192)**	**p-value**
Headache*	372/404 (92)	189/212 (89)	183/192 (95)	0.02
Chill	498/549 (91)	316/357 (88.5)	182/192 (95)	0.01
Myalgia*	230/404 (57)	75/212 (35)	155/192 (81)	<0.001
Weakness	523/549 (95)	337/357 (94)	186/192 (97)	0.19
Dizziness*	299/404 (74)	150/212 (71)	149/192 (78)	0.11
Vertigo*	112/404 (28)	59/212 (28)	53/192 (28)	0.96
Tinnitus *	83/404 (20.5)	24/212 (11)	59/192 (31)	<0.001
Anorexia	490/549 (89)	323/357 (90)	167/192 (87)	0.20
Nausea*	268/405 (66)	158/213 (74)	110/192 (57)	<0.001
Vomiting	296/549 (54)	211/357 (59)	85/192 (44)	0.001
Abdominal pain*	110/411 (27)	59/219 (27)	51/192 (26.5)	0.93
Diarrhoea	111/548 (20)	72/356 (20)	39/192 (20)	0.98
Insomnia*	313/549 (57)	203/357 (57)	110/192 (57)	0.92
Nightmare*	38/404 (9)	17/212 (8)	21/192 (11)	0.31
Palpitation*	173/404 (43)	62/212 (29)	111/192 (58)	<0.001
Dyspnea*	141/546 (26)	76/354 (21)	65/192 (34)	0.002
Cough	125/549 (23)	76/357 (21)	49/192 (25.5)	0.25
Sore throat*	49/410 (12)	29/218 (13)	20/192 (10)	0.36
Irritable	10/547 (2)	10/355 (3)	0	0.01
Rash	1/549 (0.2)	1/357 (0.3)	0	1.00
Urticaria	4/549 (0.7)	2/357 (0.6)	2/192 (1)	0.61
Itch	4/549 (0.7)	2/357 (0.6)	2/192 (1)	0.61
Chest abnormality**	2/549 (0.4)	1/357 (0.3)	1/192 (0.5)	1.00

**NOTE.** Data are shown as number (%).

* Only children aged ≥ 4 years were asked about these symptoms.

** Chest abnormality: crepitation, rhonchi.

### Fever and parasite clearance, and changes in haematocrit

At presentation, 73% of all patients were febrile, (body temperature ≥37.5 °C), while at day 2, 92% and 98% of children and adults were afebrile (p = 0.004). The proportion of patients who remained parasitaemic at day 1 after treatment was significantly higher in children (33%) compared to adults (15%) (p < 0.001, Table 3) and only one adult patient had parasites detected at day 2. The mean FCT and median PCT were significantly longer in children than in adults (Table 3). The mean haematocrit at all time points (before and after treatment) was significantly lower in children compared to adults. Baseline (day 0) and day 28 mean haematocrits improved significantly after treatment for both children and adults (Paired *t*-test p < 0.001 for both groups, Table 3).

**Table 3:** Outcome measures for the treatment of patients included in the analysis of the efficacy of artemether-lumefantrine (Coartem™) in the treatment of uncomplicated falciparum malaria in Laos

**Variables**	**Age groups**		
	**All (n = 549)**	**Children (age ≤ 15 yrs) (n = 357)**	**Adults (age > 15 yrs) (n = 192)**
42-day cure rate, no. (%) of patients ^a^	532/549 (97)	342/357 (96)*	190/192 (99)
42-day cure rate per protocol, no. (%) of patientsΨ	524/541 (97)	337/352 (96)*	187/189 (99)
Fever clearance time, mean hours (95%CI)^a,b^	25.3 (23.9-26.8)	27.6 (25.7 – 29.5)*	21.2 (19.2 – 23.2)
Patients remained febrile at day 1, no. (%)	241 (44)	169 (47)*	72 (37.5)
Patients remained febrile at day 2, no. (%)	33 (6)	29 (8)*	4 (2)
Parasite clearance time, median days (range)^a,c^	1 (1 - 3)	1 (1 - 2)*	1 (1 - 3)
Positive parasitaemia at day 1, no. (%) of patients	144/548 (26)	116/356 (33)*	28/192 (15)
Positive parasitaemia at day 2, no. (%) of patients	1	0	1
Gametocytaemia detected at anytime, no.(%) of patients	27 (5)	23 (6)	4 (2)
Gametocytaemia after treatment, no. (%) of patients	4 (0.73)	4 (1.12)	0
Gametocyte clearance time, median days (range) ^d^	7 (1 – 21)	3 (1 -21)	7 (7 – 14)
Gametocyte cleared by day 7, no. (%) ^d^	24 (89)	21 (91)	3 (75)
Median (range) gametocyte-person-weeks ^d^	0.42 (0.14 – 1.0)	0.42 (0.14 – 1.0)	0.42 (0.42 – 0.42)
*P. vivax* appearance after treatment for *P. falciparum*, no. (%) of patients	18/594 (3)	17/357 (5)*	1/192 (0.5)
Day 0 haematocrit, mean % (95%CI)	35.8(35.2 – 36.3)	33.7 (33.1 – 34.4)*	39.5 (38.7 – 40.4)
Day 1 haematocrit, mean % (95%CI)	32.4 (31.9 – 32.9)	30.4 (29.8 – 30.9)*	36.2 (35.4 – 36.9)
Day 2 haematocrit, mean % (95%CI)	31.1 (30.6 – 31.6)	29.1 (28.5 – 29.6)*	34.9 (34.1 – 35.7)
Day 3 haematocrit, mean % (95%CI)	31.1 (30.6 – 31.6)	29.0 (28.5 – 29.6)*	34.9 (34.1 – 35.6)
Day 7 haematocrit, mean % (95%CI)	33.3 (32.9 – 33.7)	31.7 (31.2 – 32.1)*	36.3 (35.6 – 37.0)
Day 14 haematocrit, mean % (95%CI)	34.4 (34.0 – 34.8)	33.3 (32.9 – 33.7)*	36.5 (35.8 – 37.2)
Day 21 haematocrit, mean % (95%CI)	35.6 (35.2 – 35.9)	34.6 (34.2 – 35.0)*	37.5 (36.8 – 38.2)
Day 28 haematocrit, mean % (95%CI)	36.2 (35.8 – 36.5)	35.3 (34.9 – 35.7)*	37.8 (37.0 – 38.5)
Day 35 haematocrit, mean % (95%CI)	36.5 (36.1 – 36.8)	35.5 (35.2 – 35.9)*	38.2 (37.5 – 38.9)
Day 42 haematocrit, mean % (95%CI)	37.6 (37.2 – 37.9)	36.7 (36.3 – 37.0)*	39.4 (38.7 – 40.1)

**NOTE.** Ψ The patient with undetermined PCR result was classified as recrudescent infection.

^a^ Intention-to-treat analysis.

^b^ Data were available from 340 and 184 patients in children and adults groups, respectively.

^c^ Data were available from 356 and 192 patients in children and adults groups, respectively.

^d^ Data were available from 23 and 4 patients in children and adults groups, respectively.

* Significant difference from the other group (p < 0.05).

### *Plasmodium falciparum* gametocyte carriage

Twenty-three (4%) patients presented with patent gametocytaemia on admission, 19 (5%) in children and 4 (2%) in adults (p = 0.07). The admission geometric mean gametocytaemia were similar for children and adults (p = 0.60). The time to clearance of gametocytes was longer in adults than in children but the difference was not statistically significant (p = 0.07). After treatment four children, but no adults, developed gametocytaemia (all at day 1) without gametocytaemia on admission. The median gametocyte-person-weeks was similar for children and adults (Table 3).

### Cure rates

Of 43 (7.8%) patients with subsequent *P. falciparum* reappearance (34 children, nine adults) PCR genotyping indicated that among these recurrent infections 16/43 (37%) [14/34 (41%) and 2/9 (22%) children and adults, respectively (p = 0.44)] had recrudescent infections. One child with *P. falciparum* reappearance had undetermined PCR result and, considering this child as having a recrudescent infection, the overall 42-day cure rates per protocol, excluding patients who were lost to follow up, withdrew consent, had persistent vomiting, or re-infection, were 97% (524/541) [96% (337/352) and 99% (187/189) for children and adults, respectively, p = 0.042]. When the patients with repeated vomiting, who were lost to follow up or were withdrawn (censored at the time last seen), considering the child with undetermined PCR result as recrudescence, and regarding recrudescent infections as failures, the 42-day cure rates (95%CI) by survival analysis (Kaplan-Meier) were 99.0% (98.3 – 99.4%) for children and 99.8% (99.0 – 99.9%) for adults. For the PCR adjusted survival analysis, the cure rates (95%CI) were 99.0% (98.3 – 99.4%) and 99.8% (99.0 – 99.9%) for children and adults, respectively. By conventional ITT analysis, the overall 42-day cure rates, adjusted for re-infection, were 97% (532/549) [96% (342/357) in children and 99% (190/192) in adults, p = 0.042]. Of 17 patients considered as having recrudescent infection (15 children and two adults), 15 (13 children and two adults) had parasite recurrence at or before day 28. Similarly, the 28-day cure rates by ITT analysis were 344/357 (96%) for children and 190/192 (99%) for adults. The median (range) interval to recrudescent falciparum infections was 21 (14–42) days. By ITT and per protocol analyses, the 42-day PCR uncorrected cure rates for children and adults were 323/357 (90%) *vs* 183/192 (95%); (p = 0.04), and 323/352 (92%) *vs* 183/189 (97%) (p = 0.02), respectively. Of 129 children aged <5 years, six had recrudescent infection and one had undetermined PCR result. If the patient with undetermined PCR result was considered a recrudescent infection, the 42-day PCR corrected cure rates among children < 5 years were 95%. There were 18 episodes of vivax malaria during the follow-up period, of which 17 were in children (p < 0.05). The median (range) interval to *P. vivax* appearance was 28 (21–42) days (Table 3).

### Factors affecting treatment response

Patients with recrudescent infections were significantly younger [median (range) age 5 (2–25) years], than those whose infections were treated successfully [median (range) age 10 (0.5–73) years, p = 0.006]. Considering the child with undetermined PCR result as recrudescent infection, the proportion of treatment failures adjusted for re-infection by PCR among children was 4.2% (15/357 patients) compared with 1.0% (2/192 patients) among adults (p = 0.042). The admission mean (95%CI) body temperature was significantly higher in patients with recrudescent infections [39.1 (38.4–39.8) ^0^ C] than those who were cured [38.4 (38.3–38.5) ^0^ C, p = 0.01]. Patients with recrudescent infections had a significantly longer mean FCT (45.0 h; 95% CI, 32.3–57.8 h) than those patients without (24.8 h; 95%CI, 23.4–26.2 h; p < 0.001). Patients with recrudescent infections also had a significantly lower haematocrit at days 3, 14, 21, 28, and 35 after starting therapy than those without recrudescence detected (p = 0.03, p = 0.01, p < 0.001, p < 0.001, and p < 0.001, respectively). The proportion of the patients reporting a past history of malaria was significantly lower in the patients with recrudescent infection (6%) compared to those without (28%) (p = 0.043). In a multiple logistic regression analysis, admission high temperature [OR = 2.2, 95%CI = 1.2 – 4.2), p = 0.01], longer fever clearance times [OR = 1.0, 95%CI = 1.0 – 1.1, p = 0.01], and lower haematocrit at day 28 [OR = 0.7, 95%CI = 0.6 – 0.9, p = 0.005] were significantly associated with treatment failure.

### Adverse events

The possible adverse events following treatment with AL are shown in Table 4. Adverse event symptoms were recorded for only those 467 (85%) patients aged ≥4 years old and able to answer questions. No serious adverse events including deaths were found during follow up. The proportion of patients with at least one recorded potential side effect after treatment was significantly higher in adults (32%) compared to children (15%) (p < 0.001), but this could be due to difficulties of obtaining histories from children. The incidence of post-treatment insomnia, weakness, anorexia, diarrhoea, nightmares and palpitation were all significantly higher in adults compared to children (p < 0.05) (Table 4).

**Table 4:** Possible adverse events (AE) found in patients included in the analysis of the efficacy of artemether-lumefantrine (Coartem™) in the treatment of uncomplicated falciparum malaria in Laos*

**Variables**	**Age groups**			
	**All (N = 549)**	**Children (age ≤ 15 years) (n = 357)**	**Adults (age > 15 years) (n = 192)**	**p-value**
At least one adverse event	114/549 (21%)	52/357 (15%)	62/192 (32%)	<0.001
Headache	32/403 (8%)	13/211 (6%)	19/192 (10%)	0.16
Insomnia	31/549 (6%)	14/357 (4%)	17/192 (9%)	0.01
Weakness	29/549 (5%)	12/357 (3%)	17/192 (9%)	0.006
Anorexia	30/549 (5%)	14/357 (4%)	16/192 (8%)	0.03
Diarrhoea	25/549 (5%)	11/357 (3%)	14/192 (7%)	0.02
Nightmare	17/403 (4%)	4/211 (2%)	13/192 (7%)	0.02
Abdominal pain	12/410 (3%)	5/218 (3%)	7/192 (4%)	0.42
Dizziness	11/403 (3%)	4/211 (2%)	7/192 (4%)	0.36
Vomiting	10/549 (2%)	8/357 (2%)	2/192 (1%)	0.32
Nausea	5/403 (1%)	2/211 (1%)	3/192 (2%)	0.67
Palpitation	5/404 (1%)	0	5/192 (3%)	0.02
Itch	5/549 (1%)	3/357 (1%)	2/192 (1%)	1.00
Vertigo	1/403 (0.25%)	0	1/192 (0.5%)	0.47
Tinnitus	1/404 (0.25%)	0	1/192 (0.5%)	0.47
Rash	1/549 (0.2%)	1/357 (0.3%)	0	1.00
Urticaria	1/549 (0.2%)	0	1/192 (0.5%)	0.35
Irritability	0	0	0	-
Dyspnoea	0	0	0	-
Hearing loss	0	0	0	-

* Symptoms are given for only those 467 patients aged ≥ 4 years old and able to answer questions about these symptoms, except that parents/guardians were asked about their charges insomnia, weakness, anorexia and itch.

## Discussion

This large clinical trial shows that artemether-lumefantrine retains excellent efficacy in the treatment of uncomplicated falciparum malaria at one site in southern Laos in both children and adults with 42-days PCR-corrected cure rates of 96% and 99%, respectively. These results are similar to those described in a comparable trial conducted in 2002-3 in nearby Phalanxay District (~60 Km west of Xepon), in which there was a 42-days cure rate of 97% in children and adults [9]. A similar trial conducted in the northern Luang Namtha Province of Laos in 2003 also showed very good efficacy and tolerability of AL with a 42-days cure rate of 94% [11]. As previously, AL was very well tolerated, proved safe and no serious adverse events were found during follow up.

Patients’ background immunity contributes substantially to the treatment response of uncomplicated malaria in Laos [6,27]. In this study, children had significantly higher recrudescence rates and *P. vivax* appearance rates than adults. A study comparing oral chloroquine and sulphadoxine-pyrimethamine for the treatment of uncomplicated falciparum malaria, before the introduction of AL, found treatment failure rates among Lao children to be 4.9 times higher than those among adults [6]. The contribution of immunity to the treatment response is also suggested by slower clearance of fever and parasitaemia among children compared to adults [1,9]. Anti-malarial drug assessments conducted only in adults, may overestimate anti-malarial efficacy risking continuation of ineffective drug regimens in national policy [28]. Anti-malarial pharmacokinetics may differ in children and but for artemether-lumefantrine these differences are small [29].

The evidence, both from a trial of artesunate monotherapy with a mean (95%CI) PCT (hours) of 23.2 (21.2-25.3) and 42-day PCR-corrected cure rate of 100% [22], and from the high efficacy of AL described here, at the same site, suggests that artemisinin resistant *P. falciparum* has not spread to this area, of southern Laos. However, regular monitoring of the clinical efficacy of artemisinin-derivatives in southern Laos, especially closer to the Cambodian border, is required.

## Conclusion

The Lao nationally recommended anti-malarial drug (artemether-lumefantrine) remains highly efficacious for the treatment of uncomplicated falciparum malaria five years after its introduction. However, regular monitoring of the efficacy of artemisinin derivatives is essential as early warning of the potential spread of artemisinin-resistant *P. falciparum* parasites.

## Competing interests

The authors declare that they have no competing interests.

## Authors' contributions

MM designed the study, recruited and followed up the patients, analysed data and drafted paper. MK and OC designed the study, recruited and followed up the patients, and revised the paper. MI designed the study, performed the molecular genetic studies, and revised the paper. TP, BH, VV, SP and NJW designed the study and revised the paper. PNN designed the study, analysed data, drafted and revised the paper. All authors read and approved the final manuscript.
